# “Treatable not curable”: trade-offs in the use of treatment-oriented language with patients who have incurable cancer

**DOI:** 10.1093/oncolo/oyae296

**Published:** 2024-11-14

**Authors:** Jason N Batten, Kristin M Kennedy, Bonnie O Wong, Stephanie A Kraft, William Hanks, David Magnus, Lidia Schapira

**Affiliations:** Department of Anesthesiology and Perioperative Medicine, University of California, Los Angeles, CA 90095, United States; Stanford University School of Medicine, Stanford, CA 94305, United States; Department of Surgery, Brigham and Women’s Hospital, Boston, MA 02115, United States; Department of Bioethics and Decision Sciences, Geisinger College of Health Sciences, Danville, PA 17822, United States; Department of Anthropology, University of California, Berkeley, CA 94720, United States; Stanford Center for Biomedical Ethics, Stanford University, Stanford, CA 94305, United States; Stanford Cancer Institute, Stanford University, Stanford, CA 94305, United States

**Keywords:** treatability statements, cancer, patient–provider communication, prognosis, end-of-life care

## Abstract

Treatment-oriented language is used by physicians to convey to patients that treatment is available for their cancer (eg, “our usual treatment for this is…,” “we can treat this,” “your cancer is still treatable”). For patients who have incurable cancer, especially for patients with a poor prognosis or who are at the end of life, it is important to understand how physicians conceptualize and use this “everyday” clinical language. We conducted a qualitative interview study with a multidisciplinary group of physicians (*n* = 30) who may care for patients with cancer at different points in their clinical course, from diagnosis to end of life. Physicians report a wide range of reasons for using treatment-oriented language in conversations with patients who have incurable cancer. However, physicians also reported concerns that this language can be ambiguous, can convey unintended positive prognostic information, and can shift attention away from important matters such as the non-curative nature of treatment or the inevitability of death. On the basis of these concerns, physicians should (1) consider whether their aims in using treatment-oriented language can be better achieved using other evidence-based communication strategies, and (2) recognize and proactively mitigate potential adverse effects of treatment-oriented language, which may manifest much later in the patient’s clinical course.

## Introduction

In conversations with patients who have incurable cancer, clinicians and patients can collude to focus on treatment while avoiding discussion of prognosis and the end of life.^1,2^ This emphasis on treatment begins with the clinicians’ use of *treatment-oriented language*, which we define here as language stating that treatment is available. Examples include a clinician explaining, “we can treat this,” describing a cancer as “treatable,” or stating, “there are things we can try.”^3^

Although treatment-oriented language cannot be avoided—patients must be informed of available treatments—it presents clinicians with a micro-ethical challenge.^4^ On the one hand, it has been described as emotional “resuscitation,” reassuring patients that something can be done during a critical time.^5^ On the other hand, simply stating that a treatment is available can lead patients to infer unintended information: that the treatment may improve prognosis, improve quality of life, or even cure the cancer.^3,6,7^

To better understand how clinicians navigate the trade-offs inherent in this everyday clinical language, we conducted a qualitative interview study that analyzed physician perspectives on the use of treatment-oriented language with patients who have incurable cancer, focusing especially on patients with a poor prognosis or who are at the end of life.

## Methods

The study was approved by the Stanford University Institutional Review Board. Using email invitations, we recruited physicians who care for patients with cancer throughout their course from diagnosis to end of life, including medical oncologists, surgeons, intensivists, and palliative care specialists.

After oral consent was obtained, audio-recorded, in-person, semi-structured interviews were conducted by a trained interviewer using a guide developed with input from a physician, an anthropologist, and a bioethicist, and refined after 3 pilot interviews. Interviews focused on physicians’ use of treatment-oriented language, using the word “treatable” as a paradigm example of this language, and asked respondents to discuss their language preferences and practices. To elicit a range of responses, physicians were queried about their and their colleagues’ use of this language, both in the context of specific scenarios of incurable cancer with poor prognosis and in their practice more generally.

Once interviewers agreed that thematic saturation had been reached, interviews were transcribed verbatim and de-identified. Two investigators coded all interview transcripts with an inductively derived codebook, which demonstrated excellent interrater reliability when tested on 25% of transcripts (Cohen’s pooled κ = 0.81). Relevant excerpts were extracted from coded data and a team-based thematic analysis was conducted to identify physician justifications for using or avoiding this language.

## Results

We interviewed 24 physicians, including 6 medical oncologists, 6 medical intensivists, and 6 surgeons whose practice ranged from surgical oncology to surgical critical care, and 6 palliative care physicians whose practice ranged from inpatient consultation to outpatient oncology clinics. Ten identified as female. The median years of practice after residency was 9 (range: 1-39). Eighteen identified as White and 6 identified as Asian.

### Rationales for using treatment-oriented language

When asked why treatment-oriented language was used in the setting of incurable cancer, physicians provided a variety of rationales (Table 1). One category focused on conveying information to patients, such as using the word “treatable” to signify (1a) the availability of treatment or (1b) the impossibility of cure. Another category focused on the management of patient emotions or decisions, such as using the word “treatable” to (2a) provide hope or (2b) encourage the patient to accept a recommended treatment. The third category was to manage conversation dynamics, such as (3a) initiating a broader discussion regarding treatment or (3b) avoiding bad news. Finally, physicians reported using treatment-oriented language (4a) to help themselves cope with difficult clinical scenarios, often by (4b) focusing on the actions physicians could take.

**Table 1.** Physician rationales for using treatment-oriented language.

Category	Rationale	Quote
1. Convey information to the patient	1a. Convey that treatment is available	[We use the word “treatable”] because it’s an accurate statement. There’s a treatment that you can offer . . . so it’s a treatable illness, so I think it’s appropriate. *D2, Medical Oncologist*
	1b. Convey the impossibility of cure	You would say something is “treatable” to convey that it’s still incurable and it’s still something that we cannot get rid of, but it’s something that we can give medications to temporarily shrink or to alleviate certain symptoms, and you know, hopefully, prolong your life—but to focus on the fact that it’s not curable. *D4, Palliative Care Physician*
2. Manage the patient’s emotions or decisions	2a. Provide hope	I want to be optimistic and hopeful, so you give them one of those little ambiguous words [“treat”] and they kind of latch on to it. *F3, Surgeon*
	2b. Encourage the patient to accept a recommended treatment	When I do use [treatment-oriented language], it’s in precisely the situation where a person feels that they have an incurable cancer and therefore there’s no treatment, so I’m quick to add, no, we have treatments for this incurable cancer. *E2, Medical Oncologist*
3. Manage conversation dynamics	3a. Start a broader conversation about treatment	[Treatment-oriented language] is a conversation starter and it can be positive and hopefully it’s followed up by a range of treatment options, and I would hope that it’s also used to convey that sometimes these treatment options that we’ve used will not be successful in prolonging your life or sometimes they will. *E4, Medical Oncologist*
	3b. Avoid discussing bad news	I think often people use the word treatable, or other similar words, because they don’t want to give bad news, and no one likes to be the bearer of bad news, and as doctors . . . you have to be the bearer of bad news, so I think they like to use the word “treatable.” *C3, Intensivist*
4. Manage physician challenges	4a. Cope with difficult clinical scenarios	I think a lot of people struggle with talking about life-and-death situations . . . Doctors are people, and a lot of doctors have problems having these discussions, and it makes it easier to compartmentalize if you start talking about diseases and not people. *F6, Surgeon*
	4b. Focus on actions physicians can take	I think that in general, oncologists never want to be the bad guys, you know, they don’t want to be the one to tell you that you have an untreatable condition and that you’re going to die, and that I had nothing to offer you. I think just we’re human at the end of the day and when we see someone suffering in front of us, we have a natural kind of feeling to want to be able to provide help for that patient . . . we want to give them assurance and hope that there is treatment for it. *E6, Medical Oncologist*

### Concerns regarding treatment-oriented language

Physicians also voiced a variety of concerns regarding treatment-oriented language (Table 2). Some concerns were related to the ability of treatment-oriented language to convey the physician’s intended meaning. Many believed that it was vague or ambiguous because (1a) it can be understood by patients in multiple ways and (1b) it is overly simplistic. Many also worried that it would actively convey unintended information, such as (2a) a favorable prognosis, including (2b) the possibility of a cure. For patients with a poor prognosis or at the end of life, physicians expressed significant concern that focusing on the treatability of cancer would shift the focus away from (3a) the intent of treatment, especially in cases of non-curative treatment, and (3b) the inevitability of death despite treatment.

**Table 2.** Physician concerns with using treatment-oriented language.

Category	Concern	Quote
1. Is vague or ambiguous	1a. Has many possible meanings	I think [treatment-oriented language is] often misused because it’s so vague that it can be interpreted in a variety of different ways. [ . . . ] I hardly ever use the word “treatable” . . . because it’s so vague it’s meaningless. *E4, Medical Oncologist*
	1b. Is overly simplistic	“There’s almost always…one more drug…and that’s the problem with modern medicine, right? So…I rarely say something in absolute that way: “treatable” is too simplistic. So then to that patient I would likely say, you know, “this is a tough tumor, but we have this thing,” in a much more concrete way, and then what the expectations of that thing is…those other terms are so absolute. *F5, Surgeon*
2. Conveys unintended information	2a. Conveys a favorable prognosis	If you used that word “treatable” [in this scenario], that would mean, yeah, we could have a positive impact on this patient: we could radiate him and make his pain better . . . but I think many patients if they hear that word “treatable” . . . would interpret that as something to do with their overall prognosis . . . I think that’s one of those tricky words. I think sometimes physicians and patients and families hear that one differently. *F3, Surgeon*
	2b. Conveys possibility of cure	I would try to steer clear of the word “treatable” because I think that it has connotations associated with it that imply a cure but . . . certainly I don’t think that’s what anyone would be saying necessarily . . . I wouldn’t say that [an incurable cancer] is treatable because unfortunately, I think “treatable” to many implies curable. *C2, Intensivist*
3. Shifts the focus of conversation	3a. Does not convey the intent of treatment	Yes, [the disease] was “treatable,” but whether people had a good enough understanding about the fact that [surgery and chemotherapy] was mostly palliative was often a gray zone . . . I think people felt like they’d gotten good treatment, but I think they had a much more sunny impression of what that meant about, you know, like what the next stage is? . . . They knew they would need later chemo, but the fact that the chemo is not very good, you know, there wasn’t a lot of focus on that part. *C1, Intensivist*
	3b. Does not address incurability and death	I think [treatment-oriented language] can really hurt [patients] in the long run . . . They have not rightfully prepared for [death] even though their physicians may have seen this coming week, months, years ahead of time. … So, I think it’s . . . devastating on multiple levels if the patients aren’t aware of the prognosis and . . . if they haven’t been spoken to truthfully. *E6, Medical Oncologist*

## Discussion

While physicians reported many reasons for using treatment-oriented language, they also harbored many concerns regarding how this language may adversely affect patients and their families. Our results do not show that treatment-oriented language is categorically “good” or “bad,” but rather highlight important communication gaps for physicians to be aware of, especially at key transitions of care such as intensive care unit admission or at the end of life.^3,6-8^ Our sample of physicians, which included those who care for patients across the clinical course, from diagnosis to end of life, allowed us to more fully explore these potential effects.

Physicians should consider whether their aims in using treatment-oriented language can be better achieved using other evidence-based strategies. For example, instead of relying on treatment-oriented language to convey hope, physicians can use silence to create space for emotional expression or explicitly discuss the patient’s hopes and fears.^4,7-9^

If physicians choose to use treatment-oriented language, they should recognize and proactively mitigate potential adverse effects that may not manifest until later in the patient’s clinical course. While this can be accomplished via sequential conversations over the course of a patient’s illness, it can also be accomplished by explicitly addressing potential ambiguities (eg, “Although we cannot cure this cancer, we have treatments that can help you feel better”).^10^

The major limitation of this study is that interview responses may not correspond to a physician’s reasoning in real-time clinical conversations. Nevertheless, interview responses reflect how physicians conceptualize the communication challenges they face and provide clarity regarding the trade-offs of using treatment-oriented language.

## Data Availability

The data underlying this article cannot be shared publicly as participants did not provide consent for the broad sharing of interview transcripts. Brief anonymized quotes will be shared upon reasonable request to the corresponding author.
